# Reciprocal Relations between Work-Related Authenticity and Intrinsic Motivation, Work Ability and Depressivity: A Two-Wave Study

**DOI:** 10.3389/fpsyg.2017.00307

**Published:** 2017-03-03

**Authors:** Astrid I. Emmerich, Thomas Rigotti

**Affiliations:** ^1^Work and Organizational Psychology, Leipzig UniversityLeipzig, Germany; ^2^Work, Organizational and Business Psychology, Johannes Gutenberg UniversityMainz, Germany

**Keywords:** authenticity, well-being, intrinsic motivation, work ability, depression

## Abstract

This study investigates the role of context-specific authenticity at work for work-related outcomes (intrinsic motivation, work ability) and depressivity. Furthermore reciprocal relations between work-related authenticity and healthy psychological functioning are investigated. Longitudinal data from 1,243 employees from 63 subsidiaries of a non-profit organization in the social sector were analyzed using multilevel structural equation modeling. Work-related authenticity at T1 predicted work ability and depressivity, but not intrinsic motivation at T2, about 6 months later. Work-related authenticity at T2 was predicted by intrinsic motivation and depressivity, but not by work ability at T1. We conclude that work-related authenticity and healthy psychological functioning are positively reinforcing each other. Thus, enabling employees to be authentic supposedly increases their well-being and is a pivotal opportunity for organizations to foster health and performance-related indicators like work ability and prevent negative health indicators like depressivity. At the same time, authenticity of employees can be fostered through workplace health promotion.

## Introduction

The European Working Conditions Survey reports that 25% of EU employees have to hide their feelings at work either all or most of the time, while 20% reported that their work sometimes or always involves tasks that conflict with their personal values (Eurofound, 2010). Even though being your “true self” has been considered a major life goal ever since ancient times (Taylor, 2003), it is not self-evident that employees can be authentic in their daily working lives (Hewlin, 2003) and experience fulfillment and self-actualisation by living out their potential (Deci and Ryan, 1985, 2000). The need to “act professional” in work settings can contradict being “real” as employees are often expected to follow role expectations and demands from supervisors, clients and colleagues that are not necessarily consistent with their feelings, values or ideas.

Authenticity has been discovered to be negatively related to anxiety (Sheldon et al., 1997; Ryan et al., 2005; Wood et al., 2008), stress (Sheldon et al., 1997; Ryan et al., 2005; Kernis and Goldman, 2006; Wood et al., 2008) and depression (Sheldon et al., 1997; Ryan et al., 2005), as well as positively linked to life satisfaction (Kernis and Goldman, 2006; Wood et al., 2008; Di Fabio and Kenny, 2016), well-being (Ryan et al., 2005; Kernis and Goldman, 2006; Robinson et al., 2013) and positive affect (Kernis and Goldman, 2006; Wood et al., 2008; Di Fabio and Kenny, 2016). This health-promoting role seems also hold true for authenticity at work. So far, studies in the work context were able to provide empirical evidence for a positive link between authenticity and work engagement (Cable et al., 2013; van den Bosch and Taris, 2013, 2014), subjective well-being (Ménard and Brunet, 2011) and positive affect (Kifer et al., 2013) as well as negative relations to burnout, stress (van den Bosch and Taris, 2014), negative affect (Kifer et al., 2013), irritation and symptoms of physical illness (Knoll et al., 2015).

In the following, we draw upon Self-Determination Theory (SDT; Deci and Ryan, 1985, 2000) to develop hypotheses about the relationship between authenticity, intrinsic motivation, work ability and depressivity at work. We suggest that authenticity at work increases intrinsic motivation as well as work ability, but also impacts well-being. We extend prior interpretations of these relationships by addressing their potential order of causality.

While there is compelling evidence about the relation between authenticity and well-being and healthy psychological functioning, to our knowledge only two studies have moved beyond a cross-sectional design (Boyraz et al., 2014; Knoll et al., 2015). For this reason, we conducted a two-wave study in order to examine relations across time—linking authenticity at work to well-being and health, but also to check for potential reciprocal effects. We will propose that not only does authenticity influence healthy psychological functioning, but that there are also reciprocal processes taking place.

In the next section, a short introduction to the concept of authenticity will be given. Then, we will explicate the proposed relationships between work-related authenticity and depressivity, intrinsic motivation and work ability based on SDT (Deci and Ryan, 1985, 2000), followed by explications for the proposed reciprocal effects.

### The concept of work-related authenticity

Authenticity can be described as “acting in accord with the true self, expressing oneself in ways that are consistent with inner thoughts and feelings” (Harter, 2005, p. 382). Drawing from historical philosophical perspectives as well as past and contemporary psychological perspectives, Kernis and Goldman (2006) developed a multicomponent conceptualisation of authenticity. This includes self-awareness which means knowing one self, e.g., one's own motives, preferences, feelings and being motivated to extend that knowledge constantly. Unbiased processing as the second component includes the relative absence of distortions in processing self-relevant information and objectivity, e.g., to positive and negative self-aspects. The third component refers to behavior that is in accordance with oneself, including one's values, preferences and needs. Relational orientation describes the relational component of authenticity and includes openness and honesty in close relationships as well as the willingness to show others one's true self. Authenticity has both trait and state components (Lenton et al., 2013; Robinson et al., 2013; Smallenbroek et al., 2016). In this study, we will investigate context-specific authenticity at work which refers to the extent that one is in tune with one's true self at work (van den Bosch and Taris, 2013). Kernis and Goldman (2006) promoted a second order factor model (based on item parcels). Hence, authenticity is considered as consisting of four interrelated but distinct components with relations that are explained by a higher order authenticity factor. Later studies mostly used authenticity as a composite measure (e.g., Ménard and Brunet, 2011; Davis and Hicks, 2013; Wickham, 2013). As we could not find solid empirical evidence on differential validity for the sub-dimensions, and we also do not see theoretical reasons for differential effects, we will formulate our hypotheses by referring to authenticity as a global construct.

### Authenticity and intrinsic motivation

Deci and Ryan (2000) propose that “intrinsically motivated behaviors are those that are freely engaged out of interest without the necessity of separable consequences” (p. 233). Self-Determination-Theory (SDT) suggests that increased intrinsic motivation, as well as social development and well-being, result from self-determined behavior. As authentic behavior has its source in the true self of an individual, authentic behavior is self-determined by nature and supposedly leads to improved intrinsic motivation, social development and well-being.

SDT explains the effect of self-determined behavior through the satisfaction of basic psychological needs: the need for autonomy, relatedness and competence (Deci and Ryan, 2000). When employees act self-determined and authentic, they seek activities that reflect their true self which ultimately fulfills their basic psychological needs and increases intrinsic motivation. The striving for, and satisfaction of, these needs results in intrinsic motivation and well-being. Van den Broeck et al. (2010) and Richer et al. (2002) were able to show that the satisfaction of these proposed basic psychological needs was related to intrinsic and autonomous motivation. In conclusion, we propose that work-related authenticity fosters intrinsic motivation:

Hypothesis 1a: There is a positive lagged effect of work-related authenticity on intrinsic motivation.

### Authenticity and work ability

Next, we propose that authenticity is not only a factor that influences the motivation to work but also the actual ability to cope with work demands and the ability to perform. The concept of work ability is grounded on the question “How good are workers at present and in the near future and how able are they to do their job with respect to work demands, health, and mental resources”? (Ilmarinen et al., 1997, p. 49). Hasselhorn and Freude (2007) describe work ability as the capability of an individual to perform well in their job at a particular time.

According to the conceptualisation of Kernis and Goldman (2006), authentic behavior is characterized by increased self-awareness and unbiased processing. Thus, authentic employees possess a more detailed, complex and accurate knowledge about their abilities and inner states regarding their work and performance. We propose that this enables them to manage themselves better regarding work demands, e.g., knowing which would be the best way for them to perform in their occupation. Furthermore, being more aware of inner states supposedly enables employees to deal with their inner states, e.g., knowing how to deal with challenging and stressful situations. In addition, unbiased processing enables employees to analyse mistakes and shortcomings in a less distorted way, which helps them to improve their abilities and performance based on that.

We further propose that when individuals act authentically at work, their work goals are in congruence with their personal goals. Sheldon and Elliot (1999) state that individuals put more effort in such self-concordant goals, which should further foster employees' work ability. Moreover, when employees are authentic in the workplace, they are supposedly spending less psychological resources on self-control or fake behaviors, e.g., through surface acting, following display rules (Grandey, 2000) or employee silence (Knoll and van Dick, 2013) which in turn leads to an increased work ability. In addition, we suggest that authentic employees benefit from increased social resources (Hobfoll, 1989) through authentic relationships at work, e.g., by the opportunity to share “actual” emotions, thoughts, perceived stress and problems (Grandey et al., 2012).

Hypothesis 1b: There is a positive lagged effect of work-related authenticity on work ability.

### Authenticity and depressivity

Another purpose we aim to investigate is whether work-related authenticity has an impact on depressivity. In order to investigate this relationship, we use non-clinical depression (Mohr and Müller, 2012) as an indicator. Non-clinical depression reflects impaired psychological well-being resulting from daily and continuous stressors (Mohr, 1986, 1991) and is based on the “cognitive triad” (Beck, 1967), a negative view of oneself, one's environment and one's future. It does not indicate a severe mental illness and is thus more useful for research in the work setting.

As previously mentioned, there are several studies that provide evidence for a relationship between general authenticity and well-being, e.g., positive mental health (Robinson et al., 2013), happiness (Wood et al., 2008), self-esteem (Kernis and Goldman, 2006; Davis et al., 2015) and decreased stress, anxiety and physical symptoms (Sheldon et al., 1997; Ryan et al., 2005). Moreover, Sheldon et al. (1997) found a negative relationship between authenticity across different social roles and depression. Theran (2011) found authenticity in relationships to be negatively related to depressivity. Based on the assumptions of SDT, we suggest that authentic self-determined behavior not only fosters positive functioning at work like work ability, but that it also has the potential to decrease depressivity. In conclusion, we hypothesize:

Hypothesis 1c: There is a negative lagged effect of work-related authenticity on depressivity.

### Reciprocal processes: the impact of intrinsic motivation, work ability, and depressivity on work-related authenticity

Though most of the studies on authenticity assume it to be a precursor for healthy functioning of individuals rather than an outcome, Wood et al. (2008) state that both causal directions between authenticity and well-being are possible. So far, only Knoll et al. (2015) and Boyraz et al. (2014) moved beyond a cross-sectional design and examined longitudinal relations. Knoll et al. (2015) found authenticity to be predictive of strain after a 6-week-interval as well as a purpose in life as an indicator for well-being to be predictive for authenticity 6 weeks later. Boyraz et al. (2014) found initial authenticity to be predictive for increased life satisfaction and decreased distress later on, yet no significant reciprocal effects were found. This indicates a need to further investigate possible reciprocal relationships. So far, we proposed a unidirectional impact of authenticity on the examined outcomes, but it is also possible that authenticity and work-related outcomes are positively reinforcing each other, resulting in a positive reciprocal circle.

Generally, the three investigated outcomes can be considered as indicators of healthy psychological functioning. Accordingly, a reciprocal effect would mean that healthy psychological functioning predicts increased authenticity. Furthermore, authenticity is one facet of eudaimonic well-being (Di Fabio and Palazzeschi, 2015; Di Fabio and Kenny, 2016). Following this idea, we would like to suggest that healthy psychological functioning can be a facilitator for being authentic.

Waterman (1993) describes eudaimonia as the result of behavior that is in accord with and toward the direction of one's true potential. In the work context, this would mean that eudaimonic well-being increases by achieving personal goals, which are in tune with one's true nature as well as from engaging in achieving goals that were difficult to accomplish and thus challenges employees to realize their full potential. The link between personal goals and eudaimonic well-being (Kiaei and Reio, 2014) as well as the link between striving for the optimal outcome and eudaimonic well-being found empirical support (Kokkoris, 2016).

We suggest the idea that intrinsic motivation, work ability and low levels of depressivity as healthy psychological functioning provide the necessary resources for engaging in personal and challenging goals. In particular, the three indicators are important because they reflect if individuals are striving for personal and challenging goals (intrinsic motivation) and if individuals have the resources to do so (work ability, low depressivity).

Thus, we hypothesize that not only does authenticity influence intrinsic motivation, work ability and depressivity, but additionally all three indicators are supposedly predictive of authenticity as a facet of eudaimonic well-being.

### Intrinsic motivation

When people act out of intrinsic motivation, they find fulfillment in those activities and enjoy doing them (Ryan and Deci, 2000). We suggest that employees who act intrinsically motivated, feel more closely connected to their work, e.g., in regard to their motives, abilities, feelings or values. Intrinsically motivated individuals were found to be more involved (Lee et al., 2011) and cognitively engaged (Walker et al., 2006) in their work. We propose that this accompanies an increased “sense of self” (Goldman, 2004) and employees are not only more aware of their inner states but also act more according to their authentic selves, increasing authenticity as a facet of eudaimonic well-being.

Hypothesis 2a: There is a positive lagged effect of intrinsic motivation on work-related authenticity.

### Work ability and depressivity

We further propose that high work ability and low depressivity positively affect eudaimonic well-being and authenticity as they reflect healthier psychological functioning to achieve personal and challenging goals.

We propose that individuals who report better health are supposedly more likely to approach challenging goals, which ultimately helps them to achieve those goals, resulting in eudaimonic well-being. Kifer et al. (2013) found a relationship between perceived power and authenticity and Satici et al. (2013) found a relationship between self-efficacy and authenticity. This supports the idea that individuals who perceive themselves as powerful are experiencing higher self-realization and eudaimonic well-being, because they are more likely to engage in challenging activities that require their maximal potential.

Thus, though not every individual that is characterized by healthy psychological functioning is automatically pursuing personal and challenging goals, it is a prerequisite to do so. Accordingly, we would like to suggest a link from high work ability and low depressivity to work-related authenticity.

Hypothesis 2b: There is a positive lagged effect of work ability on work-related authenticity.
Hypothesis 2c: There is a negative lagged effect of depressivity on work-related authenticity.

## Methods

### Respondents and procedure

The study was conducted in a German non-profit organization for education, youth and social work. The sample covered a variety of occupations, mainly jobs related to education, training and psychosocial care as well as employees in administration. The employees of the organization received an email with a link to the online survey (Unipark) and were able to answer it during their working time within 2 weeks. Employees received information about the survey. They were assured of anonymity, that participation was voluntary and that they would get a report for their location if a minimum of eight employees from an organizational unit took part.

This study was carried out in accordance with the recommendations of the Federation of the German Psychologists Association's Code of Ethics. The survey was conducted with the agreement of the workers' council of the participating organization. All subjects participated voluntarily in the survey in accordance with the Declaration of Helsinki. Ethical review and approval was not required for this study as per the institutional and national requirements.

As we were interested in mid-term effects that would indicate a sustaining effect above the short term 6-weeks effects that were found by Boyraz et al. (2014) and Knoll et al. (2015) of authenticity on well-being and vice versa, we chose an interval of 6 months between the two waves. The first measurement took place between July and September 2013, dependent on organizational considerations in the subsidiaries. The second survey (T2) was conducted between January and March 2014, approximately 6 months after T1 in each subsidiary.

The invitation to participate at the first measurement time (T1) was sent out to 2,731 employees across 64 subsidiaries, and 1,110 employees responded to the questionnaire (response rate = 40.6%). At T2, due to organizational issues, only 37 subsidiaries could be connected, comprising a total of 2.666 employees, of which 673 followed the link to the online questionnaire (response rate = 25.2%).

As we used MPlus for our analyses, we did not perform a listwise deletion, but instead made use of all information available. The analyses are based on information from *N* = 1,243 employees coming from 63 different subsidiaries. This sample for analyses consisted of more women (61.5%) than men (36.5%; 2% missing) with an average age of 44.6 years (*SD* = 11.1) at T1. On average, employees had been working in the organization at T1 for 11.4 years (*SD* = 9.0) and in their current position for 7.8 years (*SD* = 7.5). They were contracted to work on average for 32.1 h a week (*SD* = 10.1); their actual working time was 33.5 h on average a week (*SD* = 12.4). To check if there was a systematic drop out of respondents between T1 and T2, we conducted *t*-tests and Chi-Square-tests for the sociodemographic and study variables. Employees that participated only at T1 did differ from employees that took part at both measurement times in regard to working time. Participants who took part at both measurement times reported more contracted (T1 only: *M* = 31.81; T1 & T2: *M* = 33.09, *p* = 0.05) and actual (T1 only: *M* = 33.04; T1 & T2: *M* = 34.75, *p* = 0.04) weekly working hours. No systematic dropout effects were found for any of the study variables.

### Measures

#### Work-related authenticity

To measure work-related authenticity, items were adapted from the Authenticity Inventory (AI; Kernis and Goldman, 2006). The initial version consists of 45 items. The authors theoretically distinguished between four dimensions, which they labeled as awareness, unbiased processing, authentic behavior, and relational orientation. Kernis and Goldman (2006) used item-parceling to test the factor structure, and found the best fit for a second order-factor model. Subsequent research employing the AI is inconclusive concerning its factor structure: Lakey et al. (2008) as well as Gillath et al. (2010) used the four sub-dimensions, as well as a composite score without providing information on results of factor analyses within their samples. Ménard and Brunet (2011) contextualized 25 items to the working context, and constructed two dimensions labeled as Unbiased Awareness, and Authentic Behaviors. Hence, a closer look at the dimensionality of the measure seemed warranted. For our study, to create a shorter, more economic measure, five scholars from the field of work and organizational psychology were asked to rate items concerning their content validity for the four sub-dimensions, and their applicability to the working context. This resulted in 25 items (6–7 items per theoretical dimension). All of these items were translated by two people and translated back by one person (Brislin, 1986), and adapted to the working context. Due to the skewed distribution of items, we performed factor analyses on a matrix of polychoric correlations by specifying all variables as categorical and using the robust weighted least square estimator (WLSMV; Flora and Curran, 2004) in Mplus version 7.11 (Muthén and Muthén, 1998–2015). We started with an exploratory two-level factor analysis using the sample at T1 (*N* = 929 nested in *k* = 61 organizations). A parallel analysis (Horn, 1965; Liu and Rijmen, 2008; for applications with ordered categorical data see Cho et al., 2009) showed that the first four empirical eigenvalues were larger than eigenvalues derived from a random data-set. Hence, we retained four factors. The factor loading matrix revealed substantial cross-loadings of many items. In order to retain the theoretical structure of the AI, we finally chose three items with distinct factor-loadings on the four factors. For a further test of the psychometric properties we ran a set of (multilevel) confirmatory factor analyses. A one factor model [$\chi_{\left(195\right)}^{2}$ = 500.99, *p* < 0.001, *RMSEA* = 0.04, *CFI* = 0.80, *TLI* = 0.79] showed obviously a worse fit when compared to a second-order factor model [$\chi_{\left(116\right)}^{2}$ = 138.12, *p* = 0.08, *RMSEA* = 0.01, *CFI* = 0.98, TLI = 0.98]. Although a model with a correlated four factors showed even a slightly better fit [$\chi_{\left(114\right)}^{2}$ = 128.13, *p* = 0.17, *RMSEA* = 0.01, *CFI* = 0.99, *TLI* = 0.99], we used the second-order factor model in subsequent cross-lagged panel analyses to test our hypotheses, as there is no clear evidence on a differential validity of sub-dimensions. However, in addition, we will report results from exploratory analyses based on the four sub-dimensions. Sample items of the retained items are: “I am often confused about my feelings at work [recoded].” (awareness), “I often find that I am overly critical about my performance at work [recoded].” (unbiased processing), “I've often used my silence or head-nodding in a team meeting to convey agreement with someone else's statement even though I really disagree[recoded].” (authentic behavior), and “I want my colleagues to understand my weaknesses.” (relational orientation).

#### Intrinsic motivation

For assessing intrinsic motivation, the 3-item intrinsic motivation subscale from the Work Extrinsic and Intrinsic Motivation Scale (Tremblay et al., 2009) in a German version (Nestler, 2011) was used. The items ask for reasons for employees to be involved in their work, e.g., “For the satisfaction I experience when I am successful at doing difficult tasks.” Answers are given on a 7-point frequency rating scale ranging from “1- never” to “7– always/every day.” Studies confirmed the internal and construct validity of the scale. Güntert (2015), for instance, reported high positive correlations to job satisfaction [*r*_(201)_ = 0.76, *p* < 0.01], and civic virtue [*r*_(201)_ = 0.38, *p* < 0.01].

#### Work ability

Work ability was assessed with one item from the Work Ability Index (Hasselhorn and Freude, 2007). Participants were asked to imagine the highest work ability that they have ever achieved as “10” and then rate their current work ability on a scale between “1- completely unable to work” to “10– highest work ability.” The single item, when compared to the seven items instrument of work ability has shown very similar correlations to, e.g., sick leave or reports of symptoms (El Fassi et al., 2013). Ahlstrom et al. (2010) concluded: “In our study, both the WAI and the single item question strongly predicted the future degree of sick leave […]” (p. 410).

#### Depressivity

Depression in a nonclinical context was assessed with an 8-item scale by Mohr and Müller (2012). Items were answered on a 7-point frequency rating scale ranging from “1- never” to “7– almost always,” e.g., “I am in a sad mood.” A two-level confirmatory factor analyses using the eight items as indicators for a single factor showed a good fit to the data at T1 [$\chi_{\left(48\right)}^{2}$ = 107.73 *p* < 0.001, *RMSEA* = 0.04, *CFI* = 0.99, *TLI* = 0.99]. As item distributions were skewed, we treated the items to be ordinal scaled, and used the WLSMV estimation method. Mohr and Müller (2012) reported solid evidence on the validity of this measure.

## Results

Correlations of study variables at T1 and T2 are presented in Table 1. These correlations represent standardized estimates of covariation between the (latent) factors of a multilevel structural equation model. Correlations with authenticity were moderate for intrinsic motivation and work ability while being strong for depressivity. Intrinsic motivation and work ability were moderately positively related. Depressivity was moderately negatively related to intrinsic motivation, whereas it was strongly negatively related to work ability. It should also be pointed out that the factor relational orientation is mostly uncorrelated to the other dimensions, and thus seems to represent a distinct concept.

**Table 1:** **Correlations between the (latent) study variables**.

	**Variable**	**Time**	**1**	**2**	**3**	**4**	**5**	**6**	**7**	**8**	**9**	**10**	**11**	**12**	**13**	**14**	**15**
1	Authenticity	1															
2		2	0.99														
3	Awareness	1	0.93^a^	–													
4		2	–	0.99^a^	0.95												
5	Unbiased Processing	1	0.75^a^	–	0.71	0.77											
6		2	–	0.65^a^	0.55	0.75	0.90										
7	Authentic Behavior	1	0.85^a^	–	0.85	0.71	0.53	0.43									
8		2	–	0.80^a^	0.75	0.94	0.59	0.56	0.74								
9	Relational Orientation	1	0.16^a^	–	0.06^ns^	0.01^ns^	0.07^ns^	0.05^ns^	0.21	0.21^ns^							
10		2	–	0.14^a^	0.13^ns^	0.02^ns^	0.09^ns^	−0.11^**^	0.23^**^	0.03^ns^	0.75						
11	Intrinsic Motivation	1	0.44	0.57	0.41	0.62	0.28	0.25^**^	0.38	0.56	0.22	0.22^**^					
12		2	0.33	0.42	0.28	0.44	0.15^*^	0.14^**^	0.38	0.44	0.20^**^	0.28	0.79				
13	Work Ability	1	0.45	0.41	0.44	0.37	0.33	0.26	0.37	0.41^**^	0.05^ns^	0.15^*^	0.40	0.38			
14		2	0.51	0.49	0.39	0.49	0.46	0.28	0.45	0.52	0.02^ns^	0.09^*^	0.34	0.33	0.60		
15	Depressivity	1	−0.74	−0.78	−0.72	−0.74	−0.53	−0.48	−0.63	−0.77	−0.10	−0.22	−0.41	−0.50	−0.54	−0.46	
16		2	−0.72	−0.75	−0.58	−0.70	−0.65	−0.54	−0.62	−0.71	−0.07^ns^	−0.05^ns^	−0.51	−0.50	−0.57	−0.54	0.86

*N = 1243 (nested in 63 organizations). Correlations are derived from multilevel SEM models with latent variables. Correlations including the global factor Authenticity were calculated with a second order factor model. Correlations with sub-dimensions are bases on a model of intercorrelated factors*.

a *Standardized factor loadings from the second order factor model*.

T1, Time 1; T2, Time 2. All correlations (factor loadings) are significant at p < 0.001, except for those labeled with

* *p < 0.05*,

** *p < 0.01, and ns, not significant*.

In a first step we examined whether measurement invariance across T1 and T2 existed for the latent variables as a precondition for testing cross-lagged effects (Vandenberg and Lance, 2000). Several types of measurement invariance can be distinguished, but only configural (same factor pattern across time points) and metric invariance (identical loadings) are considered important for cross-lagged panel analyses (cf. Hu and Cheung, 2008). In all models we allowed measurement errors for same items to correlate between T1 and T2 (Little et al., 2007). As a simple χ2 difference test cannot be applied to categorical data, and the Satorra-Bentler correction (DIFFTEST-option in Mplus) is not available for multilevel SEM, we decided to perform the measurement invariance tests on the individual level, not considering the nested structure of the data. Results are presented in Table 2. For the measurement model of authenticity, we compared the unrestricted model to a model with invariant factor loadings for the second order factor, and also to a model with invariant factor loadings for the first and second factor. Chen (2007) recommended cut-off values of *CFI* ≤0.010 and *RMSEA* ≤0.015 (for *N* > 300) for model comparisons. Overall, the results support metric invariance of latent variables across measurement points.

**Table 2:** **Measurement invariance analyses**.

	**χ2**	***df***	***p***	***CFI***	***TLI***	***RMSEA***
**AUTHENTICITY**						
Free loadings (a)	884.30	232	0.000	0.908	0.891	0.045
2nd order Loadings invariant (b)	859.68	235	0.000	0.912	0.897	0.044
1st and 2nd order Loadings invariant (c)	850.38	243	0.000	0.915	0.903	0.042
Model difference a-b	1.41	3	0.704	0.004	0.006	0.001
Model difference a-c	7.59	11	0.750	0.007	0.012	0.003
**INTRINSIC MOTIVATION**						
Free loadings	4.72	5	0.451	1.00	1.00	0.000
Loadings invariant	10.54	7	0.160	0.999	0.998	0.020
Model difference	6.16	2	0.046	0.001	0.002	0.020
**DEPRESSIVITY**						
Free loadings	463.71	95	0.000	0.974	0.967	0.053
Loadings invariant	427.50	102	0.000	0.977	0.973	0.048
Model difference	5.98	7	0.542	0.003	0.006	0.005

*Model comparisons based on the DIFFTEST-Function in MPlus using the Satorra-Bentler correction. Df, Degrees of freedom; CFI, comparative fit index; TLI, Tucker–Lewis index; RMSEA, root-mean-square error of approximation*.

We further analyzed data with multilevel structural equation modeling using Mplus Version 7.11 (Muthén and Muthén, 1998–2015). As we sampled employees from different locations, we had a nested data structure. To account for the dependency of answers from employees working at the same site, we decided to run the analyses in multilevel-models. We treated all variables to be ordered categorical and used the robust weighted least square estimation method. To evaluate the models in terms of fit to the data, we report the comparative fit index (CFI), the Tucker-Lewis index (TLI), and the root-mean square error of approximation (RMSEA) following common research practice (cf. Coovert and Craiger, 2000).

**Figure 1:**
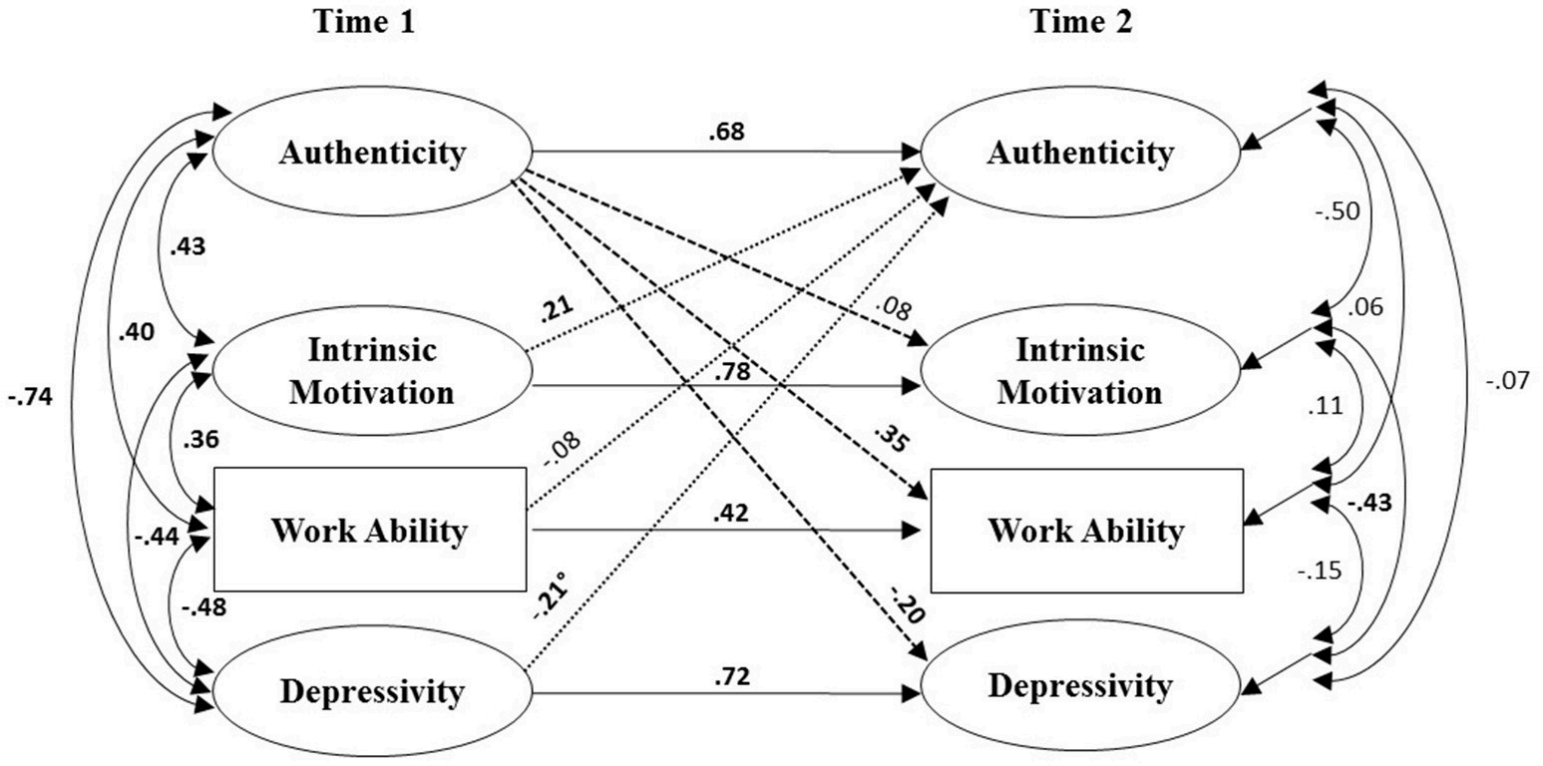
**Standardized parameter estimates of the reciprocal model with reciprocal time-lagged effects**. Dashed paths represent regular causation paths, dotted paths represent reverse causation paths; bold coefficients are significant on *p* < 0.05-level.

We started by evaluating the overall measurement model. The model that allowed all factors/constructs to covary showed a good fit to the data [$\chi_{\left(2171\right)}^{2}$ = 2362.50, *p* = 0.002, *RMSEA* = 0.01, *CFI* = 0.97, *TLI* = 0.97]. We tested the hypothesis by specifying a cross-lagged structural model that included authenticity as well as intrinsic motivation, work ability and depressivity at both measurement times (see Figure 1; Model 1, Table 3). For all models jointly including intrinsic motivation, work ability and depressivity, we allowed depressivity and work ability to covary across time points. As intrinsic motivation, work ability, and depressivity are intercorrelated, we also ran a set of models by including only one of these variables at a time (Models 2–4, Table 3). We used items as indicators for latent constructs as described in the methods section. Work ability was included as a manifest variable as it only consisted of one item. In addition, we ran exploratory cross-lagged panel models looking at the subdimensions of authenticity in isolation (Models 5–8) in order to gain information about potential differential validity of these facets.

**Table 3:** **Standardized parameter estimates of multilevel cross-lagged-panel models**.

	**Model 1 including all variables in one model**	**Model 2 intrinsic motivation**	**Model 3 work ability**	**Model 4 depressivity**	**Model 5 awareness^a^**	**Model 6 unbiased processing**	**Model 7 authentic behavior**	**Model 8 relational orientation**
	**Second order factor model of authenticity**				**Single factors of authenticity**			
	**Υ(SE)**	**Υ(SE)**	**Υ(SE)**	**Υ(SE)**	**Υ(SE)**	**Υ(SE)**	**Υ(SE)**	**Υ(SE)**
**PREDICTOR CORRELATIONS T1**								
Authenticity ↔ Intrinsic Motivation	0.43(0.03)^***^	0.44(0.03)^***^			0.38(0.04)^***^	0.28(0.04)^***^	0.41(0.03)^***^	0.21(0.03)^***^
Authenticity ↔ Work Ability	0.40(0.03)^***^		0.39(0.03)^***^		0.37(0.03)^***^	0.33(0.07)^***^	0.35(0.04)^***^	0.03(0.04)
Authenticity ↔ Depressivity	−0.74(0.02)^***^			−0.74(0.02)^***^	−0.72(0.03)^***^	−0.53(0.03)^***^	−0.64(0.03)^***^	−0.09(0.03)^***^
Intrinsic Motivation ↔ Work Ability	0.36(0.02)^***^				0.36(0.02)^***^	0.36(0.02)^***^	0.36(0.02)^***^	0.37(0.02)^***^
Intrinsic Motivation ↔ Depressivity	−0.44(0.02)^***^				−0.45(0.02)^***^	−0.45(0.02)^***^	−0.44(0.02)^***^	−0.46(0.02)^***^
Work Ability ↔ Depressivity	−0.48(0.02)^***^				−0.48(0.02)^***^	−0.48(0.02)^***^	−0.48(0.02)^***^	−0.48(0.02)^***^
**STABILITIES**								
Authenticity	0.68(0.14)^***^	0.79(0.08)^***^	0.88(0.07)^***^	0.71(0.13)^***^	0.68(0.15)^***^	0.93(0.12)^***^	0.44(0.23)^+^	0.74(0.07)^***^
Intrinsic Motivation	0.78(0.06)^***^	0.80(0.07)^***^			0.77(0.06)^***^	0.85(0.06)^***^	0.72(0.06)^***^	0.88(0.04)^***^
Work Ability	0.42(0.04)^***^		0.42(0.04)^***^		0.47(0.05)^***^	0.46(0.06)^***^	0.45(0.04)^***^	0.58(0.04)^***^
Depressivity	0.72(0.09)^***^			0.73(0.09)^***^	0.93(0.11)^***^	0.74(0.05)^***^	0.74(0.08)^***^	0.88(0.03)^***^
**REGULAR LAGGED EFFECTS**								
Authenticity (T1) → I. Motivation (T2)	0.08(0.07)	−0.02(0.09)			0.13(0.07)^+^	0.03(0.08)	0.21(0.07)^**^	0.01(0.06)
Authenticity (T1) → Work Ability (T2)	0.35(0.06)^***^		0.34(0.06)^***^		0.24(0.08)^**^	0.32(0.07)^***^	0.32(0.08)^***^	0.02(0.08)
Authenticity (T1) → Depressivity (T2)	−0.20(0.10)^*^			−0.18(0.10)^+^	0.07(0.14)	−0.27(0.07)^***^	−0.22(0.11)^*^	0.00(0.04)
**REVERSED LAGGED EFFECTS**								
I. Motivation (T1) → Authenticity (T2)	0.21(0.07)^**^	0.23(0.07)^**^			0.38(0.08)^***^	0.02(0.10)	0.21(0.12)^+^	−0.02(0.09)
Work Ability (T1) → Authenticity (T2)	−0.08(0.09)		0.01(0.09)		−0.08(0.07)	−0.12(0.22)	−0.04(0.13)	0.05(0.13)
Depressivity (T1) → Authenticity (T2)	−0.21(0.12)^+^			−0.24(0.12)^*^	−0.61(0.08)^***^	−0.04(0.10)	−0.43(0.19)^*^	−0.14(0.08)^+^
**DISTURBANCE CORRELATIONS T2**								
Authenticity ↔ Intrinsic Motivation	−0.50(0.45)	−0.16(0.32)			−0.48(0.34)	−0.53(0.57)	−0.46(0.58)	0.26(0.24)
Authenticity ↔ Work Ability	0.06(0.24)		0.07(0.19)		0.11(0.13)	−0.38(0.35)	0.21(0.31)	−0.07(0.13)
Authenticity ↔ Depressivity	−0.07(0.27)			−0.13(0.19)	−0.32(0.22)	0.30(0.52)	0.14(0.41)	0.51(0.19)^**^
Intrinsic Motivation ↔ Work Ability	0.11(0.12)				0.16(0.11)	0.24(0.14)^+^	0.04(0.11)	0.36(0.11)^**^
Intrinsic Motivation ↔ Depressivity	−0.43(0.15)^**^				−0.44(0.15)^**^	−0.61(0.17)^***^	−0.22(0.16)	−0.64(0.14)^***^
Work Ability ↔ Depressivity	−0.15(0.13)				−0.21(0.14)	−0.08(0.16)	−0.13(0.15)	−0.16(0.14)
χ2	2336.54	903.39	640.96	1621.86	891.50	890.39	891.58	994.42
(*df*)	(2171)	(820)	(607)	(1500)	(815)	(815)	(815)	(815)
*p*	0.007	0.022	0.165	0.015	0.032	0.034	0.032	0.000
RMSEA	0.008	0.009	0.007	0.008	0.009	0.009	0.009	0.013
CFI	0.977	0.977	0.984	0.980	0.991	0.990	0.990	0.976
TLI	0.976	0.975	0.983	0.979	0.990	0.990	0.990	0.974

*N = 1243, k = 63 clusters. Υ = standardized (STDYX) estimates. SE, standard error; T1, time 1; T2, time 2. RMSEA, Root Mean Square Error of Approximation; CFI, Comparative Fit Index, TLI, Tucker-Lewis Index*.

a *In Model 5, likely due to the high stability of Awareness between the two times of measurement, we modeled stability for the factor Awareness with a covariation, instead a regressive path to avoid standardized residual correlations >1; However, the general pattern of significant cross-paths was the same under both options*.

+ *p < 0.10*,

* *p < 0.05*,

** *p < 0.01*,

*** *p < 0.001*.

Results of the cross-lagged-panel analysis are presented in Table 3. The table reports correlations between latent variables at T1, stability of each construct across T1 and T2, cross-lagged effects as well as the correlations between the error terms of latent variables at T2 (disturbance correlations). Figure 1 presents standardized coefficients for the reciprocal model, based on a second order factor of authenticity. All constructs showed significant stabilities with medium (work ability) to large effect sizes. Predictor correlations at T1 were all significant on the *p* < 0.001 level and in the expected direction.

There was no significant lagged effect from authenticity to intrinsic motivation (Model 1: Υ = 0.08; *p* = 0.249; Model 2: Υ = −0.02; *p* = 0.819), which leads to a rejection of H1a. Our second hypothesis (H1b) was supported by significant cross-paths from authenticity to work ability (Model 1: Υ = 0.35; *p* < 0.001; Model 3: Υ = 0.34; *p* < 0.001). Authenticity showed a significant lagged effect to depressivity in the overall model (Model 1: Υ = −0.20, *p* = 0.038), and a marginal significant effect in the isolated model (Model 4: Υ = −0.18; *p* = 0.069). Taking the additional exploratory analyses with the single facets of authenticity into account (Model 6: Unbiased Processing, and Model 7: Authentic Behavior), this overall provides support for H1c.

Concerning our hypotheses on reversed causation, intrinsic motivation showed a significant lagged effect on authenticity (Model 1: Υ = 0.21; *p* = 0.002; Model 2: Υ = 0.23; *p* = 0.002), supporting H2a. Work ability did not show significant lagged effects on authenticity or any of its subdimensions (Model 1: Υ = −0.08; *p* = 352; Model 3: Υ = 0.01; *p* = 0.935). Hence, H2b needs to be rejected. Depressivity showed (marginally) significant lagged effects on authenticity (Model 1: Υ = −0.21; *p* = 0.071; Model 4: Υ = −0.24; *p* = 0.040).

## Discussion

The purpose of the current study was to investigate lagged reciprocal relationships between work-related authenticity and intrinsic motivation, work ability and depressivity in a sample of employees working for a non-profit organization. Results showed significant lagged effects of work-related authenticity, as a multi-dimensional, global construct on work ability and depressivity, but not on intrinsic motivation. Regarding reverse relationships, intrinsic motivation and depressivity predicted authenticity, but no significant lagged effect of work ability was found.

These results confirm and expand the empirical evidence on the relationship between authenticity and psychological health (Sheldon et al., 1997; Ryan et al., 2005; Kernis and Goldman, 2006; Wood et al., 2008; Robinson et al., 2013). We were able to contribute to previous findings by providing evidence for the idea that there are reciprocal relationships between work-related authenticity and healthy psychological functioning so that healthy psychological functioning and authenticity would positively reinforce each other. Based on our results, it can be concluded that employees that are able to live out their true self at work are expanding their ability to perform and are becoming less depressed. Moreover, increased well-being (high intrinsic motivation and low depressivity) of employees promotes their authentic behavior. The results further point out that authentic behavior at work not only benefits employees in terms of increased well-being, but also the organization as a whole as it leads to increased work ability.

These results show that work-specific authenticity is not only of importance for work-specific outcomes, but also for overall well-being. The reported lagged effects of authenticity on our proposed outcomes confirm findings from Knoll et al. (2015) and Boyraz et al. (2014) with longitudinal data where authenticity was predictive of decreased strain and distress and increased life satisfaction. In contrast, Robinson et al. (2013) did not find a significant relationship between authenticity with work colleagues and general well-being in their American and English subsample. Future studies may take a closer look at domain-specific effects of authenticity and the relation between general and domain-specific authenticity. We also know little about potential compensatory effects across life domains.

Additional exploratory analyses on the sub-dimensions of authenticity revealed that the effect of authenticity on depressivity seems mainly be attributable to unbiased processing, but also authentic behavior. An unbiased evaluation of one's self, including positive as well as negative aspects, as well as openness to personal feedback from others seems to decrease the risk for the development of depressive symptoms. Likewise, taking a stand in social situations, instead of nodding, and agreeing to decisions, which are not in accordance with own norms and values, can help to retain healthy functioning.

We found no evidence for a lagged effect of global work-related authenticity on intrinsic motivation. This conflicts with self-determination theory (Deci and Ryan, 2000), which proposes self-determined behavior to be an important predictor of intrinsic motivation. There was, however, a significant lagged effect from the sub-dimension authentic behavior to intrinsic motivation. It seems that only concrete actions that are in accordance with one's self influence intrinsic motivation. Only concrete actions can alter the social and work characteristics, which in turn promote intrinsic motivation. Authentic behavior is in contrast to “acting merely to please others, or to attain rewards” (Kernis and Goldman, 2006, p. 347), and might even be regarded a strategy of job crafting (e.g., Tims et al., 2012).

It seems important to note that the factor relational orientation, when analyzed in isolation, was neither predictive for our outcomes, nor were there significant lagged effects from intrinsic motivation, work ability or depressivity on relational orientation. It was also the subdimensions which contributed the least to the second order factor of authenticity. On a theoretical level, based on our results, relational orientation seems to be a distinct facet of authenticity that might be more relevant in regard to social relationships within the team and outcomes on team rather than individual level. In accordance with our hypotheses, there were significant lagged effects of intrinsic motivation and depressivity on work-related authenticity. Employees who act intrinsically motivated, feel more intrinsically connected to their work and thus show increased levels of true self behavior. This is interesting to note as it could imply that the awareness for one's true self could be fostered through activities that are resulting out of the intrinsic, true self of a person. Moreover, so far, most studies on authenticity implicitly assumed that authentic awareness would be leading to authentic behavior, yet we would like to raise the question if perhaps authentic behavior could lead to authentic awareness. Contrary to our hypothesis, work ability did not predict authenticity or any of its theoretical facets. Knoll et al. (2015) reported purpose in life to further authenticity. Together with our results, this supports the idea that individuals that report low levels of depressivity have the necessary resources to achieve personal and challenging goals which then fosters eudaimonic well-being. The reason why work ability showed no significant lagged effect may be that it is not only an indicator of personal resources, but also of skills and capabilities, lowering its impact on authenticity in comparison to intrinsic motivation and depressivity.

Additionally, based on the current results, we would like to propose that the processes that are influencing authenticity should not only be regarded as the result of self-determined behavior as suggested by SDT, but they should alternatively be investigated with regard to resources, e.g., in the context of the Conservation of Resources Theory (Hobfoll, 1989). For instance, authenticity might reduce resource loss through less faking behavior and less suppression of thoughts and emotions (Hobfoll, 1989).

### Limitations and future research

We want to address some limitations of the study as well as implications for further research. First, data came from a single source. Though we consider self-reports to be the most appropriate way to assess authenticity, supplemental assessment by colleagues or peers could provide additional insights (see Knoll et al., 2015). Additionally, our current findings need to be further confirmed with objective data for well-being, psychological health and performance.

In the current sample, the interval between T1 and T2 differed between, and within subsidiaries due to organizational issues which potentially has an effect on parameter estimates. Thus, it cannot be exactly stated that the occurring effects appear within a 6-month time frame. Moreover, due to relatively high stabilities of most investigated variables, longer time lags might show even stronger effects (Dormann and Griffin, 2015). Additionally, though we were able to assess cross-lagged effects with two measurement times, multiple assessments across time are necessary to assess developments and possible non-linear effects (Roe, 2014). Moreover, our data was assessed in one single company. The respondents however came from 63 different sites. We accounted for the dependency of responses of employees working at the same subsidiary by employing multilevel modeling. Though authenticity maybe is of particular importance in the non-profit and in the social sector, future studies should investigate if the impact of authenticity varies across sectors and occupations, e.g., by the extent that workers have to deal with customers, manage emotions or engage themselves personally in their work rather than “just” do their job.

We further propose that future research should examine different facets of authenticity and how they relate to outcomes like work satisfaction, well-being and performance. For example, authenticity could refer to one's values and the extent to which an employee can make decisions based on his or her values (Smallenbroek et al., 2016). Authenticity could also refer to the extent that an employee is able to show emotions, personality traits or abilities that he or she experiences as their “true self.” There are also differences in the understanding and extent of authenticity across cultures and contexts (English and Chen, 2007, 2011; Robinson et al., 2013), making cross cultural comparisons a worthwhile goal for future studies.

Our study revealed that context-specific authenticity had an impact on overall well-being and vice versa. We suggest that future research should investigate how different context-specific authenticity measures contribute to overall well-being and in which ways different well-being indicators impact context-specific authenticity measures. For instance, being authentic in one or more life domains could have buffering effects for being inauthentic in other social contexts. Alternatively, the effects of authenticity in different social roles could be additive and well-being would increase with the number of roles in which a person could be authentic. Moreover, uncertain or unfavorable working conditions might have an impact on the relation between authenticity and well-being outcomes: Davis et al. (2015) found that authenticity was a stronger predictor of self-esteem when future time was limited, considering a limited time frame as a threat to self-esteem. In the same way, authenticity as an “anchor” of one's self-worth could be a stronger predictor under working conditions that are threatening to one's self-esteem such as job insecurity, low occupational status or an effort-reward-imbalance.

Finally, future research should investigate mediating mechanisms between authenticity and healthy psychological functioning and vice versa. SDT could be used as a framework, suggesting that basic need satisfaction mediates the relationship between authentic self-determined behavior and healthy psychological functioning (Leroy et al., 2015). Findings on the relationship between authenticity and power (Kifer et al., 2013) and self-efficacy (Satici et al., 2013) go along with the idea that authentic self-determined behavior leads to an increased satisfaction of the need for competence. Conservation of Resources Theory (Hobfoll, 1989) could be used as an alternative approach as increased or decreased resources due to inauthentic behavior could mediate the relationship between authenticity and health and well-being outcomes. Moreover, we would like to suggest increased self-awareness, self-concordant goals, less pretending behavior and better social relationships as mediating mechanisms for the impact on work ability and depressivity. Further, insights about mediating mechanisms for the reversed relationships are necessary. Our suggestion that intrinsic motivation and depressivity are predictive of work-related authenticity due to the achievement of difficult and challenging goals needs to be empirically tested.

### Practical implications

Based on our results that authenticity in the workplace has a positive impact on employees' well-being, it is recommended to organizations to enable their employees to live in accord with their true selves at work. organizations should strive to enable their employees to be as authentic as possible and not require them to wear a professional mask or to conform to rigid role expectations.

At the same time, fostering well-being in organizations is beneficial for authentic behavior of employees. This is a worthwhile goal as authenticity is not only desirable for a lot of employees and would increase, e.g., their job satisfaction, but it is also necessary in various organizational contexts such as in the service sector or value-based organizations as well as in general in work contexts, e.g., in order to foster positive relationships to colleagues.

We suggest that fostering authenticity is a pivotal opportunity for organizations to conduct primary prevention in order to reduce negative health and increase work ability whereby authenticity can serve as an individual resource, profiting both individuals and organizations. Authenticity enables individuals to be aware of their selves and to critically reflect on their well-being, their performance and development. Being able to always refer back to an “inner, true self” supposedly helps individuals to manage themselves and their lives through work demands and challenges (Di Fabio and Kenny, 2016), especially in the context of the increasing flexibilities and insecurities of the modern work life inside and outside organizations (Savickas, 2011).

We propose that fostering authenticity in organizations can hardly be implemented by “technical” approaches such as setting up training and seminars but it should, rather, penetrate all other activities such as leadership (Avolio et al., 2004; Algera and Lips-Wiersma, 2012) as well as communication and everyday business (Bujisic et al., 2014). However, coaching and training could be useful for increasing the awareness about authenticity and reflecting on how to promote it in daily working life.

Moreover, we suggest that fostering authenticity is of particular importance for non-profit organizations because they are often characterized by less external rewards in comparison to for-profit organizations (Ben-Ner et al., 2011), more unfavorable working conditions (Kosny and Eakin, 2008) and because non-profits are often value- and purpose-driven. Thus, fostering authenticity is a good starting point for its employees to live out the purposes and values of their organization in their daily working lives and in order to promote well-being and health.

Our results imply that workers will be more profitable for the organization if they are “free” to be themselves. Thus, it should lead organizations to reconsider their practices, e.g., in regard to display rules, strict role expectations, autonomy and opportunities for employees to speak up (Hewlin, 2009).

According to Gable and Haidt (2005), positive psychology approaches should ultimately lead from positive individual traits and experiences to positive institutions. We suggest that the best approach to foster individual authenticity is to create environments where employees do not need to “fit in” or follow strict expectations but are rather given the autonomy to express themselves and develop a professional role that suits their identity (Hannah et al., 2011; Grandey et al., 2012; Cable et al., 2013). This will ultimately lead from authentic individuals to authentic teams and an authentic organization as a whole. An authentic organization will then in turn be perceived as genuine and trustworthy (Henderson and Edwards, 2010).

## Ethics statement

This study was conducted in accordance with the ethical standards of the Institutional and National Guidelines and with the Declaration of World Medical Association (2013). Informed consent was obtained from all individual participants included in the study. Ethical review and approval was not required for this study as per the institutional and national requirements.

## Author contributions

Study conceptualization: AE and TR. Execution of survey: AE. Analysis and write-up: AE and TR.

### Conflict of interest statement

The authors declare that the research was conducted in the absence of any commercial or financial relationships that could be construed as a potential conflict of interest. The reviewer LP and handling Editor declared their shared affiliation, and the handling Editor states that the process nevertheless met the standards of a fair and objective review.
